# Habitat heterogeneity induces rapid changes in the feeding behaviour of generalist arthropod predators

**DOI:** 10.1111/1365-2435.13028

**Published:** 2018-01-10

**Authors:** Karin Staudacher, Oskar Rennstam Rubbmark, Klaus Birkhofer, Gerard Malsher, Daniela Sint, Mattias Jonsson, Michael Traugott

**Affiliations:** ^1^ Mountain Agriculture Research Unit Institute of Ecology University of Innsbruck Innsbruck Austria; ^2^ Department of Biology Lund University Lund Sweden; ^3^ Chair of Ecology Brandenburg University of Technology Cottbus Germany; ^4^ Department of Ecology Swedish University of Agricultural Sciences Uppsala Sweden

**Keywords:** food webs, habitat complexity, habitat microstructure, molecular diet analysis, specialization index, trophic interactions

## Abstract

1. The “habitat heterogeneity hypothesis” predicts positive effects of structural complexity on species coexistence. Increasing habitat heterogeneity can change the diversity (number of species, abundances) and the functional roles of communities. The latter, however, is not well understood as species and individuals may respond very differently and dynamically to a changing environment.
2. Here, we experimentally test how habitat heterogeneity affects generalist arthropod predators, including epigaeic spiders, carabid and staphylinid beetles, under natural conditions by assessing their diversity and directly measuring their trophic interactions (which provide a proxy for their functional roles). The experiment was conducted in spring barley fields in Southern Sweden where habitat heterogeneity was manipulated by increasing within‐field plant diversity.
3. Increased habitat heterogeneity triggered rapid changes in the feeding behaviour of generalist predators characterized by lower trophic specialization at both network (H_2_’, degree of interaction specialization in the entire network) and species level (d’, degree of interaction specialization at the species level). We presume that this is because spatial separation resulted in relaxed competition and allowed an increased overlap in resources used among predator species. Predators collected from heterogenous habitats also showed greater individual‐level dietary variability which might be ascribed to relaxed intraspecific competition.
4. Our results provide conclusive evidence that habitat heterogeneity can induce rapid behavioural responses independent of changes in diversity, potentially promoting the stability of ecosystem functions.

A plain language summary is available for this article.

## 1 INTRODUCTION

The “habitat heterogeneity hypothesis” (MacArthur, 1972) states that the number of available ecological niches will increase as habitats become more complex, and that this will have positive effects on the ability of species to coexist (e.g. McClain & Barry, 2010; Stein, Gerstner, & Kreft, 2014; Tews et al., 2004). This predicted increase in the number of species and their abundances (hereafter jointly referred to as diversity) assumingly causes a range of cascading effects on ecological processes (Lovett, Jones, Turner, & Weathers, 2005), and may positively affect the stability of ecosystem functions (e.g. Cardinale et al., 2012; Hector et al., 2010; Tilman, Reich, & Knops, 2006). Support for a stabilization effect has been found, for example, in agroecosystems. Here, changes in heterogeneity due to management can be key drivers of arthropod diversity, which critically affect the delivery of ecosystem services (e.g. Haddad, Crutsinger, Gross, Haarstad, & Tilman, 2011; Langellotto & Denno, 2004; Letourneau et al., 2011).

As species within a community may not respond equally to changes in habitat heterogeneity, effects on ecosystem functions are best explained by assessing changes in their functional roles (e.g. functional traits: Gagic et al., 2015 or network metrics: Tylianakis, Laliberté, Nielsen, & Bascompte, 2010). Assessing these roles in natural systems is, however, not trivial as species, or individuals, continuously adapt to a changing environment (e.g. Ives, Gross, & Klug, 1999; Loreau & de Mazancourt, 2013; Tilman et al., 1997). These dynamics affect key components of coexistence between species such as the relative strength of intra‐ vs. interspecific competition, prey attack rate or vulnerability to enemies (Bolnick et al., 2011). Consequently, the behavioural response of whole communities to changes in habitat heterogeneity and its temporal variation is not well understood (e.g. Loreau & de Mazancourt, 2008; but see Valladares, Salvo, & Cagnolo, 2006; Foulquier, Dehedin, Piscart, Montuelle, & Marmonier, 2014). This is particularly true for highly dynamic ecosystems which undergo periodic disturbances, such as seasonal changes, floods or agricultural management (Gerisch, Agostinelli, Henle, & Dziock, 2012).

Trophic interactions can, in a network context, be a proxy for functional roles represented in communities (Heleno et al., 2014; Poisot, Mouquet, & Gravel, 2013). As such, they are very useful parameters that allow mechanistic links between habitat heterogeneity and ecosystem functions to be investigated (e.g. Pages, Gera, Romero, & Alcoverro, 2014; Tylianakis, Tscharntke, & Lewis, 2007; Vucic‐Pestic, Birkhofer, Rall, Scheu, & Brose, 2010). A small number of empirical studies has so far shown that higher habitat heterogeneity influences predator–prey interactions by reducing intraguild predation (Finke & Denno, 2002) or by strengthening dietary preferences (Birkhofer, Wise, & Scheu, 2008a; Hughes & Grabowski, 2006). However, this knowledge is primarily inferred from changes in the abundance of interacting species (i.e. from observation‐ or count‐based studies), rather than based on directly measured interactions. Capturing and quantifying these interactions as they naturally occur is urgently needed to unravel which of the potential mechanisms cause the observed changes in the functional role of predator communities (see e.g. Diehl, Mader, Wolters, & Birkhofer, 2013). This can now be done as techniques for molecular diet analyses have reached a level of detail at which trophic interaction networks can be constructed in complex multispecies systems (Clare, 2014; Traugott, Kamenova, Ruess, Seeber, & Plantegenest, 2013).

Here, we experimentally assess the effects of habitat heterogeneity by directly measuring the trophic interactions between multiple predator and prey taxa in a natural setting, including their temporal dynamics. We conducted a field experiment in cereal systems where habitat heterogeneity was manipulated by increasing within‐field plant diversity (i.e. the occurrence of arable weeds). In the resulting structure‐rich and structure‐poor habitats, we quantified both the diversity of the ground‐dwelling arthropod predator community and the individual trophic interactions of these generalist predators using novel molecular methods. Our experimental design was laid out to capture a real‐field scenario in freely developing communities. Two sampling dates were selected to reflect different levels of habitat heterogeneity due to increasing weediness in arable fields over time and to account for the phenologies and abundance dynamics of predator and prey species. The latter aspect is particularly important in our study system as, for example, cereal aphid populations (a numerically dominant herbivore prey in this system) are known to increase towards the second sampling date.

In line with the “habitat heterogeneity hypothesis,” we expected positive effects of increased complexity in structure‐rich habitats on species richness and activity density (number of specimens caught) of arthropod predators. In addition, we predicted that as habitat heterogeneity increases, trophic interaction networks will follow a similar pattern and become more complex. This prediction was originally proposed by MacArthur (1972) arguing that species will be offered a greater choice in how they respond to the environment as structural complexity increases. This may, for example, make refuges for predators more available and facilitate their coexistence by reducing negative interactions (Finke & Denno, 2002; Janssen, Sabelis, Magalhães, Montserrat, & Van der Hammen, 2007). If this prediction is supported in our study, food webs in structure‐rich habitats will be characterized by a more general network structure (Hypothesis 1). Our reasoning here is that when predators become more able to separate in space, they will also less frequently encounter one another. Consequently, they will be able to share similar prey. As at the same time refuges for prey also increase, prey may become more difficult to find for predators (Denno, Finke, & Langellotto, 2005), which should force them to more extensively explore their available niche space in search for prey. As they do so, subsets of individuals within predator species in a more complex environment will do this in slightly different microhabitats. Following this rationale, we also predict a greater variability in individual‐level predator diet (Hypothesis 2). Furthermore, these effects on the feeding behaviour of generalist predators should get more pronounced over time, as availability of ecological niches and resources increases with advancing growth of arable weeds, that is, with increasing habitat heterogeneity (Hypothesis 3).

## 2 MATERIALS AND METHODS

### 2.1 Field experiment

**Figure 1:**
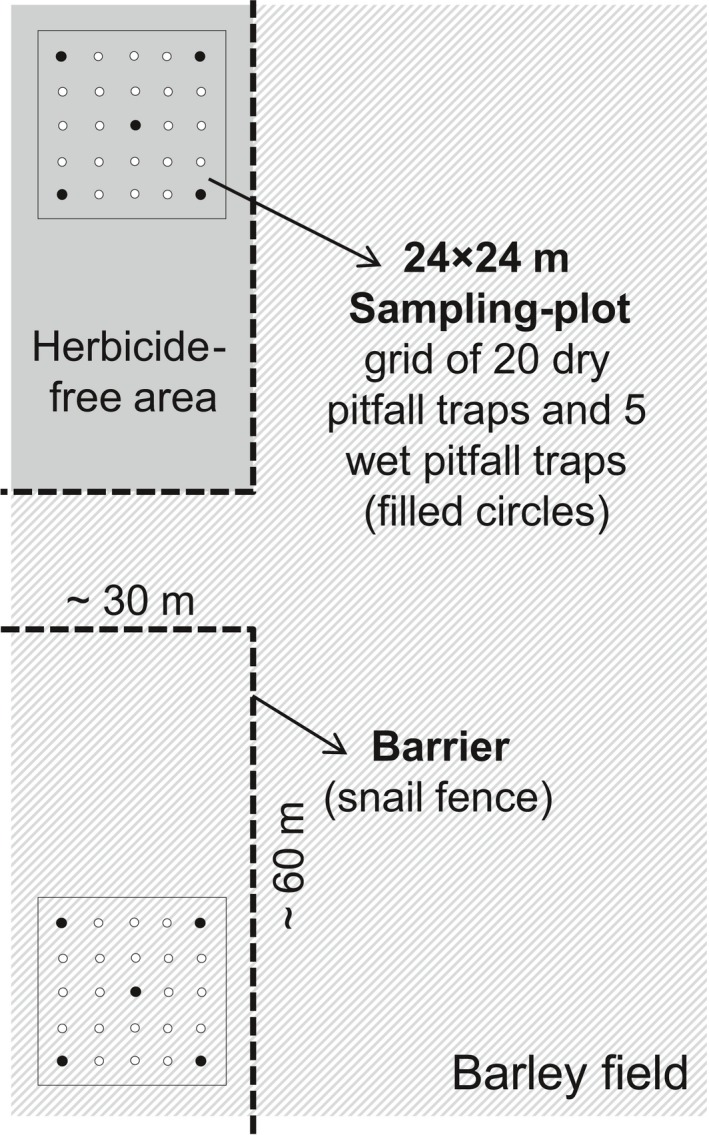
Setup of the field experiment in spring‐sown barley. Permanent installations were barriers around 30 × 60 m experimental areas assigned to two different treatments, herbicide‐free (creating structure‐rich habitats) vs. standard herbicide treatment, and grids of pitfall traps forming 24 × 24 m sampling plots

**Figure 2:**
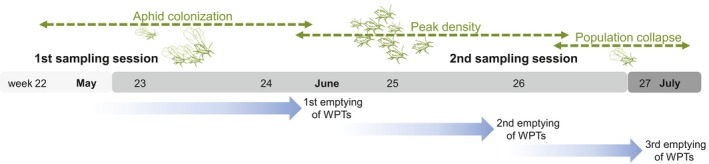
Timeline of the field experiment in spring 2012 showing from top to bottom row: aphid population phases in barley fields, two major sampling sessions (i.e. 1st and 2nd session) to assess trophic interactions of arthropod predators, extraguild prey availability, and weed development at low and high aphid densities, and three sampling periods to assess arthropod predator species richness and activity density over the three distinct phases (first activation of wet pitfall traps [WPTs] at the 1st sampling session in the respective field)

To manipulate habitat heterogeneity, four conventional spring barley (*Hordeum vulgare*) fields located in Southern Sweden (Scania) were chosen for the experiment: field 1 (N56° 11.19425’ E13° 5.49467’; 51 m a.s.l.), field 2 (N56° 6.97797’ E13° 6.786’; 35 m a.s.l.), field 3 (N55° 49.28393’ E13° 41.53262’; 136 m a.s.l.), and field 4 (N56° 1.76755’ E12° 51.84857’; 49 m a.s.l.). The barley was sown in April 2012. At two opposing sides of each field, experimental areas of *c*. 30 × 60 m were established and randomly assigned to two different treatments. One area was not treated with herbicides, allowing arable weeds to grow, whereas herbicides (sprayed during the last 2 weeks of May) were applied to the remainder of the field, including the second area (Figure 1). Weed species were identified and their percentage ground cover was estimated within four 1 m^2^ areas in each sampling plot (see below; Appendix S1: Figure S1‐1 in Supporting Information). In all four fields, herbicide application lead to significantly lower weed ground cover (2.3% ± 1.9% [*M* ± *SD*, 1st sampling session; see below, Figure 2] and 2.0% ± 1.8% [2nd session] vs. 16.3% ± 10.5% [1st session] and 30.2% ± 23.6% [2nd session]). We assume that herbicide application primarily affected arthropods indirectly through changes in habitat heterogeneity resulting from reduced weed cover (e.g. Nyffeler, Dean, & Sterling, 1994). Direct lethal effects by herbicides on arthropods such as spiders are usually weak (Baines, Hambler, Johnson, Macdonald, & Smith, 1998; Haughton, Bell, Boatman, & Wilcox, 2001; Michalková & Pekár, 2009), although behavioural changes may occur shortly after herbicide exposure. In a recent study, Korenko et al. (2016) exposed wolf spiders to several common herbicides and found that fresh residues reduced predator activity during 4 hr after exposure, but except for one herbicide that is not registered in Sweden (Basta 15) no effects on activity were detected with 48‐hr‐old residues. Therefore, since sampling of predators in our experiment was conducted several days after herbicide application, we assume that such direct sub‐lethal effects were of minor importance. Both experimental areas within each field were fenced no later than 5 days after spraying, in order to constrain movement of ground‐dwelling arthropod predators from the surrounding field. This approach ensured that the measured trophic interactions occurred within the respective treatments (Figure 1). For fencing, a dedicated snail fence (PET; EXCOLO^®^ GmbH, Vreden, Germany) was buried in the soil with *c*. 15 cm of the fence above the soil level. Vegetation along the fence was removed on a regular basis to avoid it to become overgrown (see Appendix S1: Figure S1‐2). Within each 30 × 60 m experimental area, a 24 × 24 m sampling plot was established with a buffer zone of 3 m to the nearest edge/fence. Twenty‐five pitfall traps (plastic cups, Ø 11.5 cm, 11 cm depth; Figure 1) were buried at ground level to form a grid in each sampling plot, with 4 m spacing between traps. A metal roof was installed above each trap to protect trap content from rain and debris (see Appendix S1: Figure S1‐2).

In each sampling plot, all sampling, except wet pitfall trapping (see below), was conducted during two major sessions: at a time of low aphid densities (1st session starting week 22 [30th May to 6th June]; aphid colonization phase) and *c*. 1 month later (2nd session starting week 25 [23th to 29th June]; peak density phase) when high aphid densities were expected (Figure 2). Because each sampling session was completed within 24 h per field, all four fields were sampled within 1 week. During the fieldwork, care was taken to disturb the communities within the sampling plots as little as possible (e.g. minimum walking distances between traps).

Twenty pitfall traps in each grid lacked trapping fluid (dry pitfall traps; Figure 1) in order to collect live arthropod predators at 24‐hr intervals during the two major sampling sessions (i.e. samples to assess trophic interactions, see below; Figure 2). These traps were partly filled with clay balls to provide structure and shelter for the collected arthropods and to reduce in‐trap predation (Sunderland, Powell, & Symondson, 2005). On the evening preceding each sampling session, all dry pitfall traps were activated in the respective field. Each trap was then left open for a 24‐hr interval (evening to evening) and emptied twice after *c*. 12 and 24 hr. At each session, additional hand collections were conducted at ten random patches of Ø 30 cm (sampling time: 3 min) per sampling plot. Smaller and/or less mobile arthropod species (e.g. staphylinids, linyphiids), which may otherwise be underrepresented in pitfall traps (Sunderland et al., 2005), were carefully picked by hand. Each collected predator was individually placed in a 2 ml reaction tube, immediately cooled at 3–5°C and freeze‐killed at −50°C on the same day to prevent DNA degradation.

The remaining four corner traps and the single centre trap in each grid contained trapping fluid (wet pitfall traps; Figure 1). These five traps were half‐filled with saturated saline solution as preservative and a drop of odour‐free detergent (to reduce the surface tension) and were operated for 4–5 weeks. Wet pitfall traps were emptied three times during this period to allow the assessment of arthropod predator communities over time. These sampling intervals coincided with important periods of aphid population dynamics in the fields: colonization, peak density and population collapse (Figure 2). Arthropod predators caught in wet pitfall traps were stored in 70% ethanol and identified to species level (ground beetles [Carabidae] and spiders [Araneae]); rove beetles (Staphylinidae) were identified to genus level. The combined approach with both wet and dry pitfall traps was necessary, as individuals from wet pitfall traps cannot be subjected to molecular diet analyses due to high risk of contamination (i.e. regurgitates from one predator contaminating others as they drown; King, Read, Traugott, & Symondson, 2008). Dry pitfall traps, on the other hand, would not provide reliable information about activity densities if operated unattended over longer sampling periods (mortality, predation, escape risk).

To assess the availability of extraguild prey at the two major sessions in each sampling plot, (1) aphids were counted on 50 randomly selected barley tillers, that is, grass stems (except for site 3 at the 2nd sampling session, where aphid numbers were very high and only 25 tillers were examined), (2) earthworms were counted in 10 randomly distributed soil samples (20 × 20 cm, depth *c*. 10 cm), and (3) springtails were caught with 20 sticky traps, consisting of brown paper cards (10 × 5 cm; Raupenleimpapier; Stähler Austria GmbH & Co. KG, Graz, Austria) sprayed with aerosol glue (Insekten‐Fangleimspray; F. Schacht GmbH & Co. KG, Braunschweig, Germany). Each sticky trap was anchored horizontally to the ground in the vicinity of dry pitfall traps and was active during the 24‐hr dry pitfall trapping interval (see Appendix S1, Figure S1‐2). Sticky traps were recollected and stored at 4°C until morphological classification of springtails as either Arthropleona or Symphypleona.

Data loggers (Tinytag Ultra 2; Gemini Data Loggers Ltd., West Sussex, UK) were placed in the centre of each sampling plot to measure air temperature in the crop over each 24‐hr dry pitfall trapping interval. During the field experiment, air temperature increased from 10.3°C ± 4.8°C (*M* ± *SD*, 1st sampling session) to 14.6°C ± 4.2°C (2nd session) in all four fields, and no significant differences in temperature were found between fields or treatments.

### 2.2 Molecular diet analysis

All ground‐dwelling arthropod predators (i.e. carabid and staphylinid beetles, lycosid, linyphiid, and other spiders) collected from dry pitfall traps or active hand collections were morphologically identified to the lowest taxonomic level possible (in most cases species) and thereafter subjected to DNA extraction. Whole animals were processed according to the protocol described in Staudacher, Jonsson, and Traugott (2016) which allows the extraction of any prey DNA present in the predator's intestinal tract. All extractions were done in a separate pre‐PCR laboratory; negative controls (lysis buffer) were included within each batch of 96 samples and tested with universal COI primers to check for DNA carry‐over contamination during all steps.

Predator DNA samples were screened with the three diagnostic multiplex PCR assays: “MPI,” “MPII beetles/thrips,” and “MPII spiders” presented in Staudacher et al. (2016) (see Appendix S2: Figure S2‐1). In particular, all samples were first screened for DNA of different extraguild and intraguild prey groups (i.e. aphids, earthworms, springtails, dipterans, beetles/thrips, spiders and lacewings) using the “MPI” assay. Primer pairs that target the consumer DNA (i.e. beetle or spider DNA) amplified an internal control which allowed checking for false negatives. In cases where no amplicons could be detected in this first screening, predator samples were re‐tested with universal COI primers and five samples where no DNA could be amplified at all were excluded from the final dataset.

Predator DNA samples were further screened with the MPII assays: all beetles with “MPII beetles/thrips” and all spiders with “MPII spiders” to assess intraguild predation (i.e. beetle–beetle and spider–spider trophic interactions). Note that in this step, the primer pair targeting the genus/family of the respective predator examined was excluded.

In addition, spiders testing positive for “beetle” prey in the group‐specific “MPI” were further tested with “MPII beetles/thrips” to identify these prey types to a lower taxonomic level. Likewise, beetles testing positive for “spider” prey were tested with “MPII spiders.” Note that of 29 beetles testing positive for “spider” prey, 25 could not be assigned to a specific spider taxon in the “MPII spiders,” suggesting that feeding interactions occurred with spider taxa other than the targeted genera (for details on coverage, see Staudacher et al., 2016). To resolve this issue, these samples were additionally subjected to DNA barcoding and 15 samples could then be assigned, mostly to Linyphiidae and Lycosidae (see Appendix S2: Protocol S2‐2).

Positive (artificial mixes of target DNA at low concentrations) and negative controls (PCR‐grade water instead of DNA) were run within each 96‐well PCR plate to check for correct amplification and DNA carry‐over contamination. All PCR products were separated and visualized using the QIAxcel electrophoresis system (Qiagen, Hilden, Germany) following the protocol described in Staudacher et al. (2016).

### 2.3 Data handling and statistical analysis

#### 2.3.1 Arthropod predator community (wet pitfall traps; three sampling periods)

Species richness and activity density of ground‐dwelling arthropod predators were calculated separately for each sampling plot and sampling period.

#### 2.3.2 Extraguild prey availability (two major sampling sessions)

As aphid counts at the 1st sampling session (aphid colonization) were naturally low and included a high number of zeros, these were analysed as presence or absence of aphids on tillers. At peak density phase, aphids could be analysed based on counts; one field (site 1) was excluded from this analysis, as aphid populations had already collapsed in that field prior to the 2nd sampling session. Earthworm numbers were generally low and, therefore, analysed as presence or absence of earthworms in soil samples. Springtail abundances as estimated with sticky traps were analysed based on counts.

#### 2.3.3 Predator trophic interactions (dry pitfall traps, hand collections; two major sampling sessions)

Trophic interactions were assessed using diagnostic PCR assays designed for arthropod predator–prey systems in cereals and covering all major prey groups (see Staudacher et al., 2016). The proportion of predators testing positive for a specific prey (i.e. prey DNA detection rate) in such a screening for multiple different prey types provides a reliable proxy for predation rates (Symondson, 2012). Note that for arthropods, post‐feeding prey DNA detection intervals span *c*. 3–4 days (e.g. Sint, Raso, Kaufmann, & Traugott, 2011) and that a predator can test positive for more than one prey type. From all predator individuals that tested positive for DNA of at least one of the targeted prey taxa, detection rates were analysed for aphid prey as well as pooled non‐aphid extraguild (EGP: earthworms, springtails, dipterans, thrips) and intraguild (IGP: beetles, spiders, lacewings) prey groups. For the latter two, detection rates were calculated as the presence or absence of any prey detection within the respective group to facilitate the interpretation of results. Likewise, this grouping of prey items was used for calculation of d’ (specialization index, see below). In all other calculations, resolution was as per the molecular assays (for targeted prey taxa, see Appendix S2: Figure S2‐1).

For all analyses that took predator taxa into account, rare taxa were pooled on a higher level (such that congenerics were grouped by their respective genus) or excluded as they occurred in very low numbers, were only caught in a single sampling plot, or tested negative in the molecular screening (for details, see Appendix S3: Table S3‐1b).

The software “Food Web Designer” (Sint & Traugott, 2016) was used to graphically represent trophic interaction networks for each treatment and sampling session. Network specialization metrics were calculated separately for all combinations of fields, treatments and sampling sessions to avoid potential problems associated with aggregated networks. For comparison of treatment effects at the network level and at the species level, the H_2_’ index of network specialization and the d’ index of specialization were calculated, respectively (Blüthgen, Menzel, & Blüthgen, 2006). Note that both H_2_’ and d’ are bound between 0–1, with 1 representing complete specialization, suggesting at the network level that each predator would only feed on a single prey taxon, or at the species level that a described prey species was only consumed by a single predator taxon.

The diet composition of predators was analysed using PERMANOVA models, with treatment and sampling session as fixed factors, based on 9,999 permutations (Anderson, 2001) and Bray‐Curtis similarities (Legendre & Legendre, 1998). Targeted prey taxa with fewer than three detections across all predator taxa were excluded from this analysis (i.e. prey taxa “dipterans,” “lacewings” and *“Pterostichus”*; and in addition, *“Harpalus”* and *“Pachygnatha”* at the 1st sampling session). Permutational analysis of multivariate dispersion (PERMDISP2) was used to test the effects of treatment and sampling session on the dietary variability of each predator taxon (9,999 permutations; Anderson, Ellingsen, & McArdle, 2006). A graphical representation of differences in diet composition was provided by non‐metric multi‐dimensional scaling (NMDS) ordination, upon which the effect of treatment has been superimposed as a standard ellipsoid area (95% CI).

In cases where regression‐based tests were performed, the best fitting model was selected based on Akaike's information criterion (AIC or AICc to correct for small sample sizes). All models were tested with treatment and date (sampling period or sampling session) or an interaction term between the two included as fixed factors, and field was always included as a blocking factor to minimize residual variation. For each model, diagnostic plots were examined to check whether model assumptions were met (Zuur, Ieno, & Elphick, 2010). For all univariate count data, generalized linear models (GLMs) were fitted with Poisson distributions or negative binomial distributions when data were overdispersed. Presence/absence data were tested using GLMs with binomial error distribution. As both H_2_’ and d’ are indexes bound between 0 and 1, these were tested using Beta regressions (BR) which share properties with conventional linear models but constrain predicted values to fall between 0 and 1 (Cribari‐Neto & Zeileis, 2010).

All analyses were performed in r version 3.1.2 (R Core Team, 2017) using packages “vegan” (Oksanen et al., 2016), “mass” (Venables & Ripley, 2002) and “betareg” (Cribari‐Neto & Zeileis, 2010) for statistical analyses and model validation; package “bipartite” (Dormann, Fründ, Blüthgen, & Gruber, 2009) was used to derive network metrics.

## 3 RESULTS

A total of 3,849 ground‐dwelling arthropod predators were collected from the wet pitfall traps during the three sampling periods, representing 33 carabid species, nine staphylinid genera, and 49 spider species (Appendix S3, Table S3‐1a). Predator species richness and activity density (Appendix S3, Table S3‐2) were significantly higher at aphid peak density than at aphid colonization or the population collapse phase (GLM_richness_: *z* = 4.16, *p* < .001; GLM_activity density_: *z* = 3.21, *p* = .001). Variables were not, however, significantly affected by habitat heterogeneity.

During the colonization phase, aphid densities were significantly lower in the structure‐rich habitats (GLM: *z* = −3.61, *p *< .001; proportion of tillers with aphids 0.21 vs. 0.35). Aphid numbers did not differ significantly during aphid peak density phase when the number per tiller had increased to 34.5 ± 26.8 (*M* ± *SD*) and 35.4 ± 31.3 in the structure‐rich and structure‐poor habitats, respectively. Earthworm densities were low and they were not significantly affected by habitat heterogeneity or sampling session. In the structure‐rich habitats, 2.2 times higher abundances of Symphypleona (springtails) were recorded (GLM: *z* = 1.65, *p* = .010).

**Figure 3:**
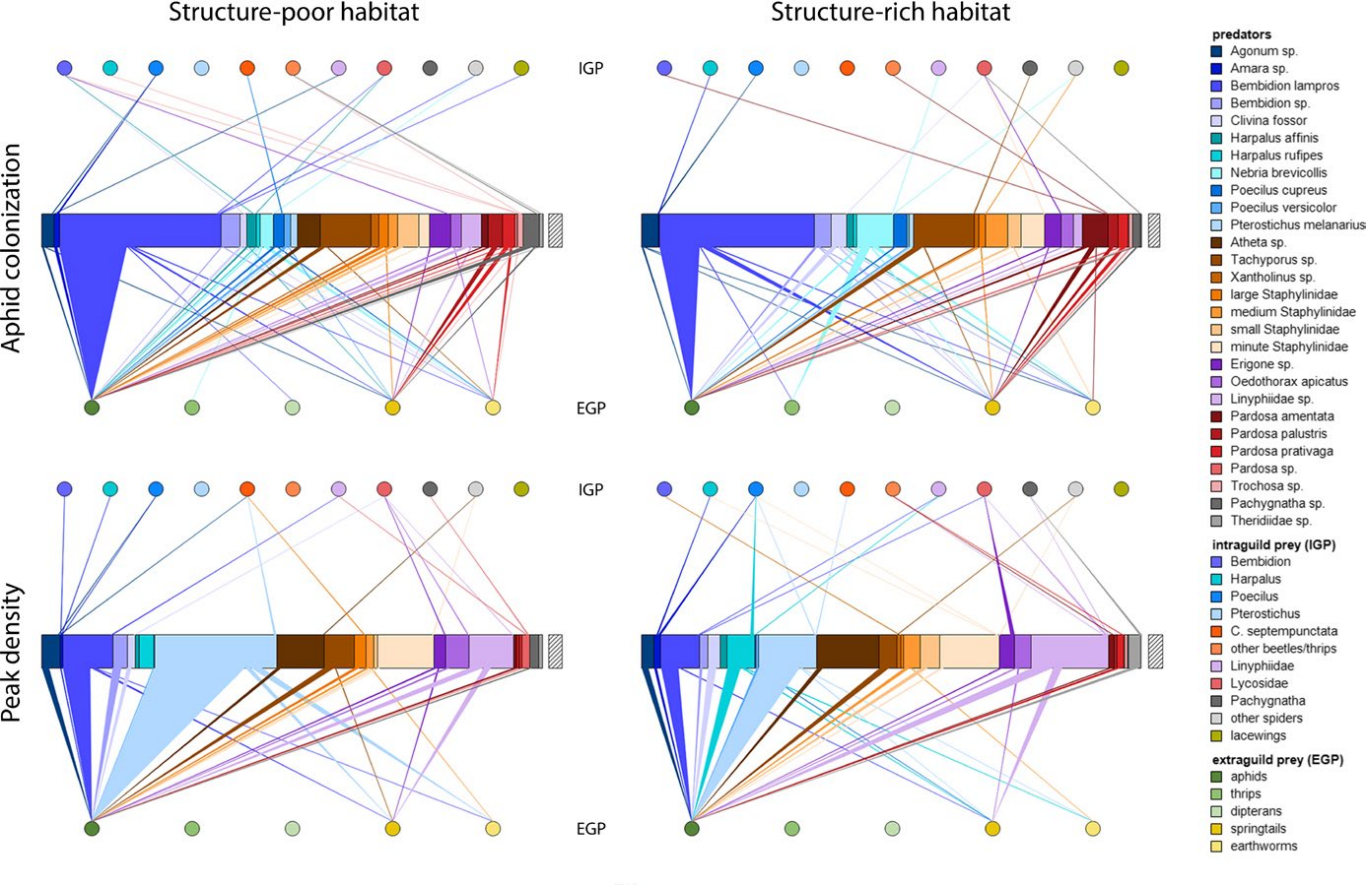
Trophic interaction networks between arthropod predators and prey taxa in barley fields at aphid colonization (upper panel) and peak density (lower panel) phase (networks result from pooling data of all four fields per treatment, i.e., structure‐rich and structure‐poor habitats). Trophic links to extraguild (circles below bars, EGP) and intraguild (circles above bars, IGP) prey are represented as triangles; the width of the base of each triangle represents the proportion of individuals within a predator taxon testing positive for specific prey taxa. The right offset bar represents 10 predator individuals each. Note that the predator taxa *Agonum* sp. comprises both *Agonum muelleri* and *Anchomenus dorsale* (formerly *Agonum dorsale*). See also Appendix S3: Table S3‐1b for details on taxonomic assignment and grouping of predator taxa

Among the 1,641 molecularly screened predators (for complete list, see Appendix S3: Table S3‐1b), 759 tested positive for DNA of at least one of the targeted prey taxa. The DNA detection rate for aphid prey was generally high and increased significantly towards the aphid peak density phase in both structure‐rich and structure‐poor habitats (GLM: *z* = 3.98, *p* = .008; Figure 3). Detection rates of non‐aphid EGP were significantly higher at aphid colonization phase (GLM: *z* = −2.667, *p* = .008) and in the structure‐rich habitats (GLM: *z* = 3.83, *p* < .001). Intraguild predation was generally low with no significant differences between treatments or sampling sessions.

**Figure 4:**
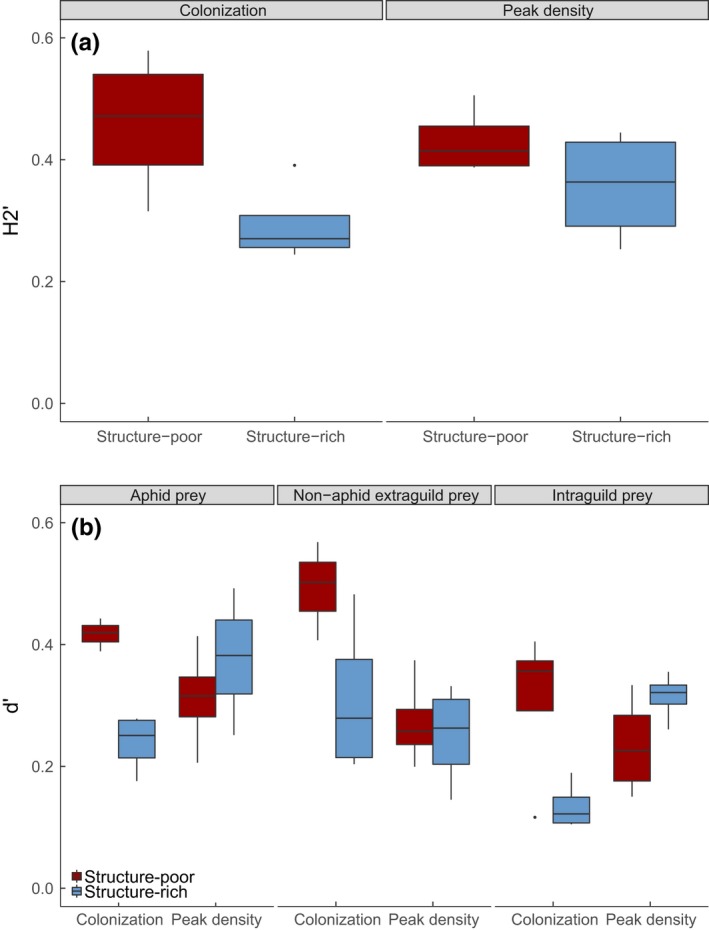
(a) Network‐level (H_2_’) and (b) species‐level (d’) specialization of trophic interaction networks in barley fields at aphid colonization and peak density phase (four fields pooled for structure‐poor and structure‐rich habitats). Note that the species‐level specialization is shown for aphids and pooled non‐aphid extraguild (earthworms, springtails, dipterans, thrips) and intraguild (beetles, spiders, lacewings) prey groups. The midline of the boxplot represents the median, with the upper and lower limits of the box being the third and first quartile, respectively. Whiskers will extend up to 1.5 times the interquartile range from the top/bottom of the box to the furthest datum within that distance; data beyond that distance (outliers) are represented individually as points

Network‐level specialization (H_2_’) was significantly lower in the structure‐rich habitats (BR: *z* = −4.65, *p* < .001). A significant interaction term between treatment and time (BR: *z* = 2.3, *p* = .022) showed that differences due to habitat heterogeneity were less pronounced towards the phase of aphid peak density, with networks in the structure‐poor habitats becoming less specialized and networks in the structure‐rich habitats becoming more specialized with time (Figure 4a).

In accordance with this, species‐level specialization (d’) between aphid prey and predators showed that a wider range of predators consumed aphids in the structure‐rich than in the structure‐poor habitats (BR: *z* = −4.8, *p* < .001). Treatment did, however, interact with time (BR: *z* = 3.49, *p* < .001), with the two habitats becoming more similar towards the aphid peak density phase. Specialization on non‐aphid EGP was lower at the phase of aphid peak density (BR: *z* = −3.93, *p* < .001) and in the structure‐rich habitats (BR: *z* = −3.33, *p* < .001). Similarly, specialization on IGP was lower in the structure‐rich habitats (BR: *z* = −3.86, *p* < .001), but treatment again interacted with time indicating less pronounced differences towards aphid peak density phase (BR: *z* = 4.11, *p* < .001) (Figure 4b).

**Figure 5:**
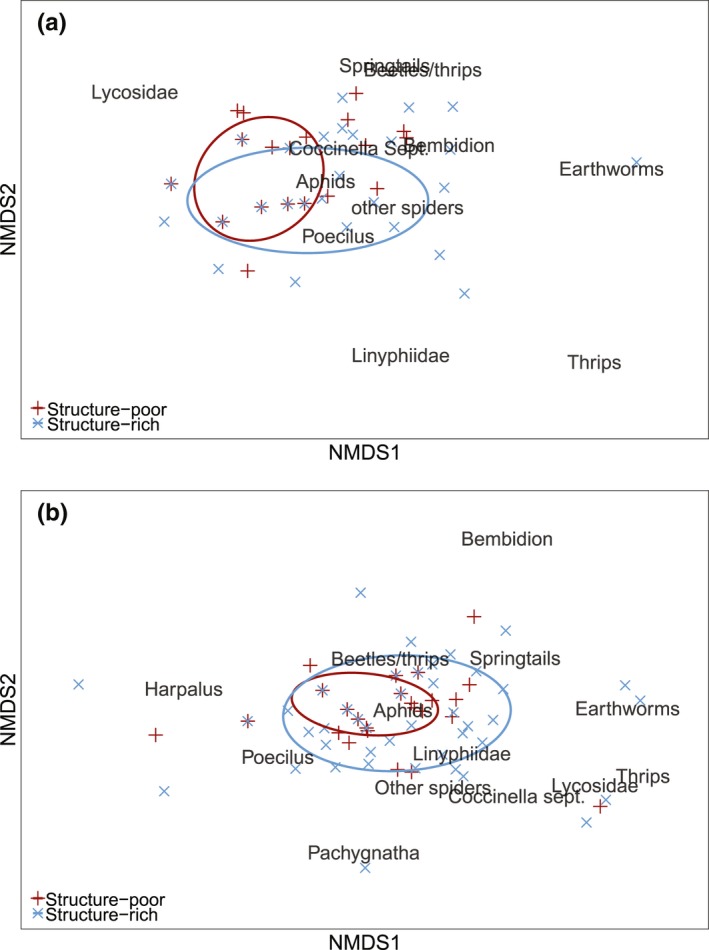
Non‐metric multi‐dimensional scaling (NMDS) ordination of the arthropod predator diet composition in spring barley fields at (a) aphid colonization (2‐d stress 0.099) and (b) peak density phase (2‐d stress 0.11). Resemblance in diet composition between predator taxa from structure‐poor (red) and structure‐rich (blue) habitats are shown as symbols, and standard ellipsoid areas represent the 95% confidence interval of treatments’ centroids. Note that “beetles/thrips” denotes for “other beetles/thrips”

Neither diet composition nor individual‐level dietary variability between predators was significantly affected by habitat heterogeneity at aphid colonization phase (Figure 5a). Towards peak density phase, individual‐level variability in predator diet was, however, significantly higher in the structure‐rich habitats (PERMDISP2: *F*_1,101_ = 5.08, *p* = .028) (Figure 5b).

## 4 DISCUSSION

**Figure 6:**
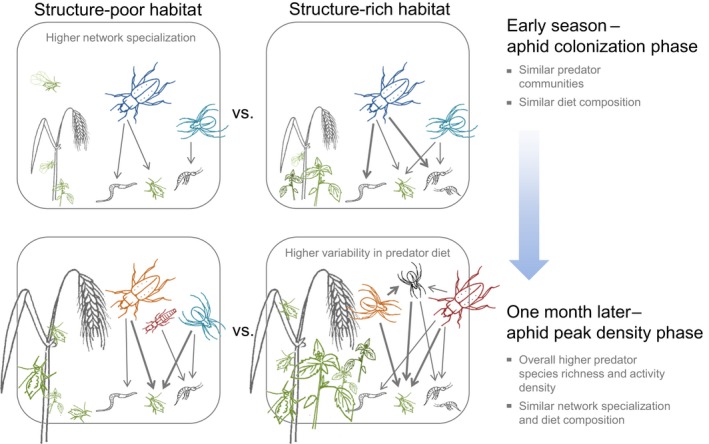
Graphical summary of the main findings showing that even if habitat heterogeneity does not affect the diversity of arthropod species in a periodically disturbed system, it can trigger immediate behavioural responses in generalist predators. Early on in the season, predators in structure‐rich compared with structure‐poor habitats were interacting with a greater diversity of prey species as well as were more likely to share these with other predators (i.e. more general trophic network structure). This changed later in the growing season when food was easier to find. Then, instead subsets of individuals within each species started to focus on slightly different prey, depending on which sub‐part of the habitat those individuals happened to be living in (i.e. greater variability in individual‐level predator diet). The four boxes represent sampling plots in structure‐poor vs. structure‐rich habitats in spring barley fields at aphid colonization (upper panel) and peak density phase (lower panel). The strength of trophic interactions between predator and prey is indicated by the width of the connecting arrows

Our results show that increasing habitat heterogeneity alters the functional role of arthropod predator communities and that these short‐term behavioural changes are not accounted for by altered diversity (Figure 6). Increasing habitat structure leads to both a more general trophic network structure (supporting Hypothesis 1) and a greater variability in individual‐level predator diet (supporting Hypothesis 2). Whereas the effect on the latter did get more pronounced over time, the effect on arthropod food web specialization decreased with advancing habitat heterogeneity (partly supporting Hypothesis 3).

Contrary to our expectations, we found no immediate effects of habitat heterogeneity on either species richness or activity density of ground‐dwelling arthropod predators. This result highlights the importance of considering diversity components beyond the number of predator species and their abundances (as only recently emphasized by Birkhofer, Diekötter, Meub, Stötzel, and Wolters (2015) and Gagic et al. (2015)). Especially in habitats characterized by periodic disturbance (e.g. annual crops), effects on diversity may not be easily detected (e.g. Bengtsson et al., 2003; Tscharntke, Klein, Kruess, Steffan‐Dewenter, & Thies, 2005). We show, however, that the species present in an ecosystem can within a short time respond to changing environments by adjusting their trophic interactions (see also Tylianakis et al., 2007). At the aphid colonization phase (i.e. shortly after inducing different levels of within‐field plant diversity in cereal systems), specialization at both network (H_2_’) and species level (d’) was significantly lower in structure‐rich than structure‐poor habitats. These patterns result from an increased overlap in the use of available food resources among arthropod predators in structurally complex environments. An explanation for this is that in these more stratified habitats, which have a greater range of microhabitats, predators are less likely to encounter one another. As suggested by Finke and Denno (2002), this makes predators compete less strongly and thus be less selective, facilitating a greater range of trophic interactions. For the same reasons, it is likely that prey refuges also become more available, which may, as prey becomes more difficult to find, have a similar effect in that they induce predators to be less selective. This has implications in, for example, agricultural systems where soil tillage or harvesting periodically will change the habitat of predators. When this occurs, predator communities are likely to be negatively affected as competition will increase with lost structure. In such cases, increasing habitat structure afterwards may benefit predator communities, which will affect the provision of ecosystem services (e.g. biocontrol: Birkhofer et al., 2008b; Finke & Snyder, 2008; Langellotto & Denno, 2004).

About a month later, during aphid peak density phase, variability in individual‐level predator diet was significantly higher in structure‐rich compared with structure‐poor habitats. This indicates that trophic interaction networks were becoming more flexible, that is, on average arthropod predators had a less fixed food web position, which contributed to the effect of the increased overlap in resource use between species due to the enhanced heterogeneity in structure‐rich habitats. Araújo, Bolnick, and Layman (2011) predicted a similar response to a release from intraspecific competition which occurred in the cereal systems studied when aphids were present in large numbers. One community‐level implication that might arise from this is that the food web position of species in structurally complex environments becomes less fixed with an increased individual‐level variation. Because of this, species would be more plastic and able to adapt more easily when facing ecological changes. Additionally, as this added variability would allow species to overlap more in their ecological roles, this should increase redundancy and contribute to stabilizing ecosystem functions (e.g. Boeye, Kubisch, & Bonte, 2014; Bolnick et al., 2011; Haddad et al., 2011; Ives et al., 1999).

The 3rd hypothesis stated that as differences in habitat heterogeneity between structure‐rich and structure‐poor habitats increase over time, any effects we detected on the feeding behaviour of generalist predators should also get more pronounced. Our results do partly support this hypothesis: while at aphid peak density phase the diet became more variable between predators at the individual level (i.e. developing over time), differences in the trophic specialization (H_2_’ and d’) did attenuate from the first to the second sampling date. Early in the season, when the most common herbivore prey in the system (i.e. aphids) was still limited, higher heterogeneity in structure‐rich habitats enabled predators to feed more on shared prey groups, such as springtails. About a month later, when habitats were more heterogenous and aphids were a highly abundant and easy to find prey, intraspecific competition for prey between predators relaxed. This allowed individual predators to be less restricted to their optimal prey choice, and explore new opportunities within the subsets of the habitat each species was inhabiting (Araújo et al., 2011; Finke & Snyder, 2008). Our study provides empirical support for both mechanisms, that is, increased overlap in resource use and relaxed intraspecific competition, inducing the finally observed changes in the functional roles of natural arthropod communities that are facing increased habitat heterogeneity.

## 5 CONCLUSION

The current findings show that even if habitat heterogeneity does not affect the number of arthropod species and their abundances in a periodically disturbed system, it can trigger an immediate behavioural response in generalist predators: trophic specialization decreased at both network (H_2_’) and species level (d’). Later in the growing season, when levels of intraspecific competition were presumable low due to high herbivore prey availability, diet variation at the individual level was more pronounced in structure‐rich compared with structure‐poor habitats. Our results provide conclusive evidence that habitat heterogeneity can induce rapid behavioural responses before changes in diversity may even manifest itself, potentially promoting the stability of ecosystem functions.

## AUTHORS’ CONTRIBUTIONS

M.T. and M.J. obtained funding and conceived/designed the study together with K.S. and K.B. K.S., O.R.R. and G.M. accomplished field work/collected samples (with support of field assistants), G.M. identified specimens (morphologically), and K.S. performed laboratory work (with support of laboratory assistants). O.R.R., K.S. and D.S. analysed the data/compiled tables and figures. K.S. and O.R.R. wrote the first draft of the manuscript, and M.T., K.B., D.S. and M.J. contributed to finalizing the paper. All authors gave final approval for publication.

## DATA ACCESSIBILITY

Data deposited in the Dryad Digital Repository https://doi.org/10.5061/dryad.n4120 (Staudacher et al., 2017).
