# Supplementary material for: Habitat heterogeneity induces rapid changes in the feeding behaviour of generalist arthropod predators
Source: Funct Ecol. 2018 Jan 10;32(3):809–19. doi: 10.1111/1365-2435.13028 (PMC5887929; doi:10.1111/1365-2435.13028)
Supplement: Supplementary file 1 [file FEC-32-809-s001.pdf]

Functional Ecology
BRITISH ECOLOGICAL SOCIETY

Habitat heterogeneity induces rapid changes in the feeding behaviour of generalist arthropod predators

Karin Staudacher, Oskar Rennstam Rubbmark, Klaus Birkhofer, Gerard Malsher, Daniela Sint, Mattias Jonsson & Michael Traugott

The habitat heterogeneity hypothesis predicts that complex ecosystems will be inhabited by a more diverse set of species than simple ones. Moreover, increased habitat heterogeneity can affect the functioning of communities, but this is not well understood as species and individuals may respond differently and dynamically to a changing environment. In this study we used a DNA-based approach to examine how predatory beetles and spiders change their feeding behaviour in response to changes in habitat heterogeneity in cereal fields. With a wider range of plants, such as weeds, the habitat becomes more spatially complex. When this happens, shelter for both prey and predators may become easier to find. Consequently, predators no longer have to fear encountering each other as much as in more simple settings. At the same time they are forced to explore their habitat more in search of food. We found that not only does the feeding behaviour of predators change quickly after plant diversity is increased, this occurs before any differences are seen in predator richness. Early on in the season, predators in structure-rich compared to structure-poor habitats became more likely to interact with more prey species as well as sharing these with other predators. This

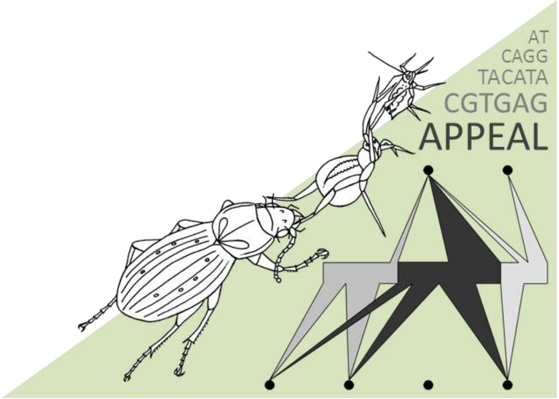

Artwork by Karin Staudacher

changed later in the growing season when food was easier to find. Then, subsets of individuals within each species started to focus on slightly different prey, depending on which part of the habitat those individuals happened to be living in. This shows an example of how habitat structure affects how strongly predators compete for prey. This has implications for agriculture, where increasing habitat heterogeneity could be used to make pest control more reliable by allowing a greater suite of predator species to contribute to this service.
