# Supplementary material for: Habitat heterogeneity induces rapid changes in the feeding behaviour of generalist arthropod predators
Source: Funct Ecol. 2018 Jan 10;32(3):809–19. doi: 10.1111/1365-2435.13028 (PMC5887929; doi:10.1111/1365-2435.13028)
Supplement: Supplementary file 2 [file FEC-32-809-s002.pdf]

Supporting Information

APPENDIX S1

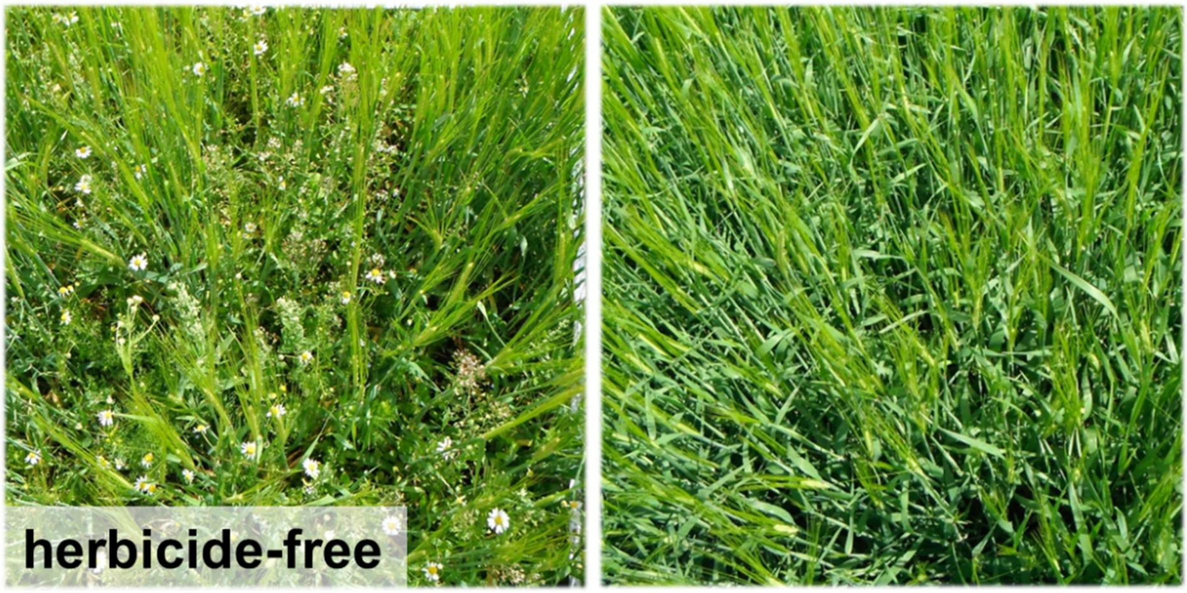

Figure S1-1 Field experiment in cereal systems in Southern Sweden (Scania). Barley and weed plants in sampling-plots assigned to two different treatments, herbicide-free (i.e., creating structure-rich habitats; left) and herbicide (right). The most abundant weed taxa were *Chenopodium album*, *Capsella bursa-pastoris*, *Polygonum aviculare*, *Tripleurospermum* sp., and *Stellaria media* and grasses of the genera *Phleum*, *Poa,* and *Festuca*. Photos display 1 m² areas to estimate ground cover at the 2nd sampling session (i.e., aphid peak density phase) in June 2012.

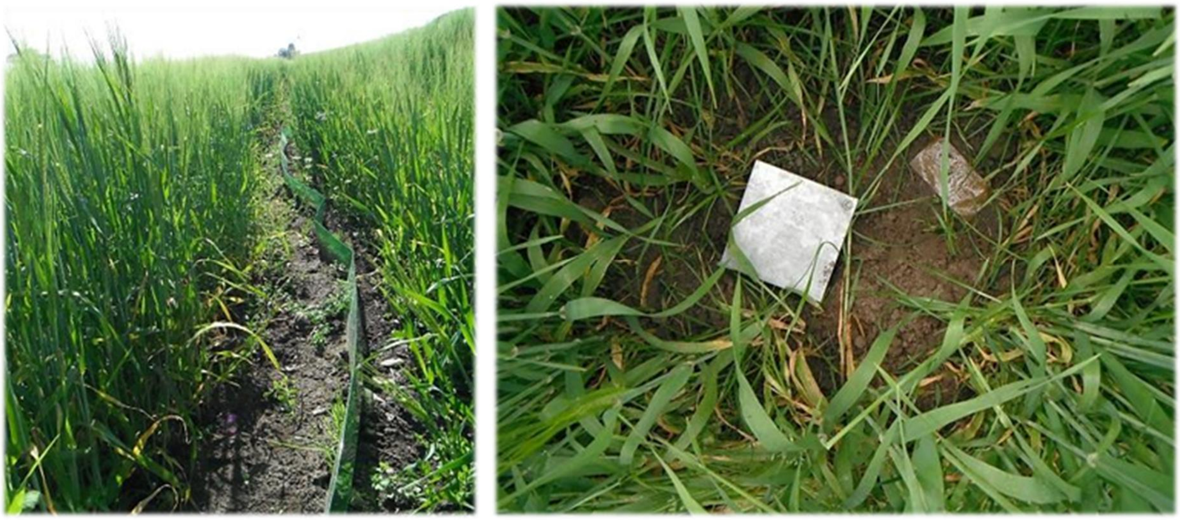

Figure S1-2 Pictures from the field experiment: barrier (snail fence) around 30×60 m experimental area (left) and dry pitfall trap with metal roof and sticky trap right beside it (right).
