# Supplementary material for: Habitat heterogeneity induces rapid changes in the feeding behaviour of generalist arthropod predators
Source: Funct Ecol. 2018 Jan 10;32(3):809–19. doi: 10.1111/1365-2435.13028 (PMC5887929; doi:10.1111/1365-2435.13028)
Supplement: Supplementary file 3 [file FEC-32-809-s003.pdf]

Supporting Information

APPENDIX S2

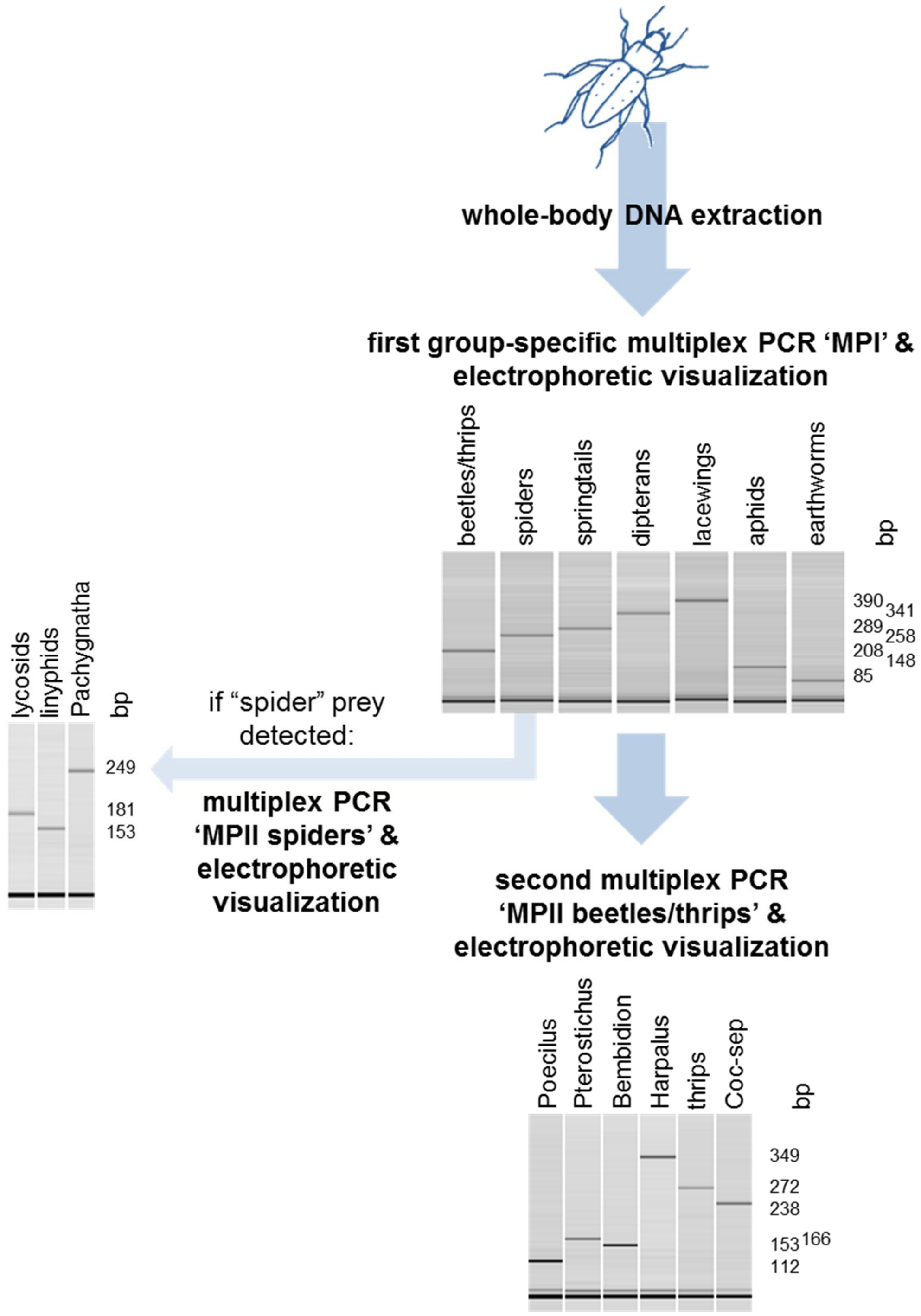

Figure S2-1 Molecular gut content analysis workflow: screening procedure using the example of a carabid beetle. For details see Staudacher, Jonsson & Traugott (2016).

Protocol S2-2 Additional DNA barcoding approach for beetles testing positive for 'spider' prey DNA.

Singleplex PCR using the group-specific 'spiders' primer pair

The 'MPI' PCR protocol (Staudacher et al. 2016) was employed with minor modifications: amplification was performed in a total volume of 10 µl containing 2.2 µl of DNA extract, 1× QIAGEN Multiplex PCR Master Mix (Qiagen, Hilden, Germany), 0.5 µM of each primer (S407 and A408; targeting 18S spider DNA), 0.5× Q-solution (Qiagen), 5 µg BSA, and 30 mM TMAC (Sigma-Aldrich, St. Louis, USA). The thermocycling program was 95°C for 15 min, 35 cycles of 94°C for 30 s, 62°C for 90 s, and 72°C for 90 s, and finally 72°C for 10 min. PCR products were separated and visualized using the automated capillary electrophoresis system QIAxcel (Qiagen).

DNA sequencing and BLAST

Sequencing of purified PCR products with the forward 'spiders' primer S407 was conducted by Eurofins MWG Operon (Ebersberg, Germany). Generated 18S sequences were edited manually using BioEdit Sequence Alignment Editor v7.1.9 (Hall 1999) and matched with the GenBank sequence database using BLAST (Basic Local Alignment Search Tool). All barcoded 'spider' prey samples were assigned to family level to make results comparable to the ones of the 'MPII spiders' assay. Note that the semi-conserved nature of the 18S gene is generally not suited for species-specific assignment of taxa; moreover, for lycosid/gnaphosid/clubionid spiders' current availability of published reference sequences is too low for reliable identification of taxonomic levels lower than family.

Staudacher, K., Jonsson, M. & Traugott, M. (2016) Diagnostic PCR assays to unravel food web interactions in cereal crops with focus on biological control of aphids. *Journal of Pest Science*, **89**, 281–293.

Hall, T.A. (1999) BioEdit: a user-friendly biological sequence alignment editor and analysis program for Windows 95/98/NT. *Nucleic Acids Symposium Series*, **41**, 95–98.
