# Supplementary material for: Habitat heterogeneity induces rapid changes in the feeding behaviour of generalist arthropod predators
Source: Funct Ecol. 2018 Jan 10;32(3):809–19. doi: 10.1111/1365-2435.13028 (PMC5887929; doi:10.1111/1365-2435.13028)
Supplement: Supplementary file 4 [file FEC-32-809-s004.pdf]

Supporting Information

APPENDIX S3

Table S3-1 Complete list of arthropod predator taxa collected during May/June 2012 in barley fields in Southern Sweden (Scania).

a Predator taxa from wet pitfall traps which were used for community assessment (i.e., species richness and activity density). The most abundant taxa with proportions >5% of the total catch are indicated by *.

order	family	species
Coleoptera	Carabidae	*Agonum assimile*
		Agonum muelleri
		Amara communis
		Amara familiaris
		Amara similata
		Amara spreta
		Anchomenus dorsale
		Bembidion aeneum
		Bembidion guttula
		Bembidion lampros
		Bembidion obtusum
		Bembidion quinquestriatum
		Bembidion tetracolum
		Calathus fuscipes
		Calathus melanocephalus
		Carabus granulatus
		Carabus nemoralis
		Clivina fossor
		Demetrias atricapillus
		Harpalus affinis
		Harpalus rufipes
		Loricera pilicornis
		Nebria brevicollis
		Notiophilus germinyi
		Poecilus cupreus
		Poecilus lepidus
		Poecilus versicolor
		*Pterostichus melanarius**

		Pterostichus niger
		Pterostichus nigrita
		Trechus discus
		Trechus micros
		Trechus quadristriatus
	Staphylinidae	*Anotylus* sp.
		Atheta sp.
		Mycetoporus sp.
		Philonthus sp.
		Quedius sp.
		Stenus sp.
		Tachinus sp.
		Tachyporus sp.*
		Xantholinus sp.
		Staphylinidae (not ident.)
**Araneae**	Lycosidae	*Pardosa agrestis*
		*Pardosa amentata**
		Pardosa lugubris
		Pardosa paludicola
		Pardosa palustris
		Pardosa prativaga
		Pardosa pullata
		Pirata piraticus
		Trochosa ruricola
		Trochosa sp.
		Trochosa spinipalpis
		Trochosa terricola
		Troxochrus scabriculus
		Lycosidae (juv.)*
	Linyphiidae	*Agyneta rurestris*
		Araeoncus crassiceps
		Araeoncus humilis
		Bathyphantes gracilis
		Bathyphantes parvulus
		Dicymbium nigrum
		Diplocephalus cristatus
		Diplocephalus latifrons
		Diplostyla concolor
		*Erigone atra**
		Erigone dentipalpis
		Erigone longipalpis
		Leptorhoptum robustum
		Oedothorax agrestis

	*Oedothorax apicatus**
	Oedothorax fuscus
	Oedothorax retusus
	Pocadicnemus pumila
	Porrhomma pygmaeum
	Savignya frontata
	Tenuiphantes tenuis
	Linyphiidae (juv.)
Tetragnathidae	*Pachygnatha clercki*
	*Pachygnatha degeeri**
	Pachygnatha listeri
Clubionidae	*Clubiona neglecta*
	Clubiona reclusa
Gnaphosidae	*Drassyllus pusillus*
	Gnaphosidae (juv.)
Theridiidae	*Achaearanea riparia*
	Robertus arundineti
	Theridion impressum
	Theridion sisyphium
	Theridion sp.
Thomisidae	*Ozyptila praticola*
	Ozyptila trux
	Xysticus audax
	Xysticus cristatus
	Xysticus kochi
	Xysticus ulmi

b Predator taxa from dry pitfall traps/hand collections which were used for molecular diet analysis (number of specimens analysed per taxon indicated in parentheses). Last column displays the taxonomic assignment of taxa when grouped for statistical analysis (specialization indices, diet composition) and graphical representation of trophic interaction networks (Fig. 3). Note that a few predator taxa were not subjected to these calculations as they occurred in very low numbers, were only caught in a single sampling-plot, or tested negative in the molecular screening.

order	family	species	grouping for calculations
**Coleoptera**	Carabidae	*Acupalpus meridianus* (1)	
		Agonum muelleri (14)	*Agonum* sp.
		Anchomenus dorsale (33)	*Agonum* sp.
		Amara aenea (3)	*Amara* sp.
		Amara eurynota (1)	*Amara* sp.
		Amara familiaris (3)	*Amara* sp.
		Amara plebeja (1)	*Amara* sp.
		Amara similata (3)	*Amara* sp.
		Amara sp. (1)	*Amara* sp.
		Bembidion lampros (325)	*Bembidion lampros*
		Bembidion aeneum (7)	*Bembidion* sp.
		Bembidion guttula (3)	*Bembidion* sp.
		Bembidion obtusum (18)	*Bembidion* sp.
		Bembidion tetracolum (18)	*Bembidion* sp.
		Calathus fuscipes (1)	
		Calathus melanocephalus (10)	
		Carabus granulatus (1)	
		Carabus nemoralis (2)	
		Clivina fossor (34)	*Clivina fossor*
		Demetrias atricapillus (2)	
		Harpalus affinis (21)	*Harpalus affinis*
		Harpalus distinguendus (1)	
		Harpalus rufipes (37)	*Harpalus rufipes*
		Loricera pilicornis (3)	
		Nebria brevicollis (43)	*Nebria brevicollis*
		Notiophilus aquaticus (1)	
		Notiophilus germinyi (1)	
		Poecilus cupreus (21)	*Poecilus cupreus*
		Poecilus versicolor (10)	*Poecilus versicolor*
		Pterostichus diligens (1)	
		Pterostichus melanarius (144)	*Pterostichus melanarius*
		Pterostichus niger (5)	

		Synuchus vivalis (3)	
		Trechus micros (1)	
		Trechus quadristriatus (2)	
		Trechus secalis (1)	
	Staphylinidae	*Anotylus rugosus* (4)	
		Atheta gregaria (63)	*Atheta* sp.
		Atheta sp. (35)	*Atheta* sp.
		Philonthus sp. (11)	
		Quedius sp. (2)	
		Stenus sp. (1)	
		Tachinus sp. (10)	
		Tachyporus sp. (128)	*Tachyporus* sp.
		Xantholinus sp. (13)	*Xantholinus* sp.
		large Staph. (not ident.) (24)	large Staphylinidae
		medium Staph. (not id.) (45)	medium Staphylinidae
		minute Staph. (not id.) (115)	minute Staphylinidae
		small Staph. (not id.) (45)	small Staphylinidae
		very large Staph. (not id.) (3)	
		Staph. (not id.) (1)	
**Araneae**	Lycosidae	*Alopecosa cuneata* (2)	
		Pardosa amentata (34)	*Pardosa amentata*
		Pardosa palustris (24)	*Pardosa palustris*
		Pardosa prativaga (25)	*Pardosa prativaga*
		Pardosa agrestis (5)	*Pardosa* sp.
		Pardosa sp. (5)	*Pardosa* sp.
		Trochosa ruricola (6)	*Trochosa* sp.
		Trochosa sp. (3)	*Trochosa* sp.
	Linyphiidae	*Erigone atra* (35)	*Erigone* sp.
		Erigone dentipalpis (16)	*Erigone* sp.
		Erigone sp. (juv.) (1)	Linyphiidae sp.
		Oedothorax apicatus (49)	*Oedothorax apicatus*
		Oedothorax fuscus (1)	Linyphiidae sp.
		Oedothorax retusus (1)	Linyphiidae sp.
		Oedothorax sp. (3)	Linyphiidae sp.
		Agyneta rurestris (7)	Linyphiidae sp.
		Araeoncus humilis (5)	Linyphiidae sp.
		Bathyphantes gracilis (24)	Linyphiidae sp.
		Savignya frontata (2)	Linyphiidae sp.
		Tenuiphantes tenuis (3)	Linyphiidae sp.
		Linyphiidae (not id.) (66)	Linyphiidae sp.
	Tetragnathidae	*Pachygnatha clercki* (1)	*Pachygnatha* sp.
		Pachygnatha degeeri (18)	*Pachygnatha* sp.
		Pachygnatha sp. (8)	*Pachygnatha* sp.
	Clubionidae	*Clubiona reclusa* (1)	

	Clubionidae (not id.) (1)	
Dictynidae	Dictynidae (not id.) (1)	
Gnaphosidae	Gnaphosidae (not id.) (1)	
Theridiidae	*Achaearanea riparia* (3)	Theridiidae sp.
	Enoplognatha ovata (1)	Theridiidae sp.
	Neottiura bimaculata (1)	Theridiidae sp.
	Robertus arundieti (1)	Theridiidae sp.
	Robertus lividus (1)	Theridiidae sp.
	Theridion impressum (4)	Theridiidae sp.
	Theridion sp. (3)	Theridiidae sp.
	Theridiidae sp. (2)	Theridiidae sp.
Thomisidae	*Xysticus kochi* (1)	

Table S3-2 Arthropod predator community in Swedish barley fields in spring 2012. Columns show the richness (R; number of species for carabids and spiders, genera for staphylinids) and the activity density (AD; number of specimens caught) of predators collected in wet pitfall traps over three time periods in sampling-plots assigned to structure-poor and structure-rich habitats (fields pooled).

	**structure-poor habitat**						**structure-rich habitat**					
	aphid colonization		peak density		population collapse		aphid colonization		peak density		population collapse	
	R	AD	R	AD	R	AD	R	AD	R	AD	R	AD
carabids	14	83	23	227	19	177	17	102	18	208	14	130
staphylinids	6	74	9	213	7	101	8	119	10	150	8	64
spiders	26	365	27	362	19	215	28	398	30	573	22	288
**TOTAL**		**522**		**802**		**493**		**619**		**931**		**482**
